# Machine learning predicts improvement of functional outcomes in spinal cord injury patients after inpatient rehabilitation

**DOI:** 10.3389/fresc.2025.1594753

**Published:** 2025-08-25

**Authors:** Mohammad Rasoolinejad, Irene Say, Peter B. Wu, Xinran Liu, Yan Zhou, Nathan Zhang, Emily R. Rosario, Daniel C. Lu

**Affiliations:** ^1^ Department of Neurosurgery, David Geffen School of Medicine, University of California, Los Angeles, CA, United States; ^2^ Department of Molecular, Cell and Developmental Biology, University of California, Los Angeles, CA, United States; ^3^ School of Medicine, University of Colorado Anschutz Medical Campus, Aurora, CO, United States; ^4^ Department of Computer Science, University of California, Los Angeles, CA, United States; ^5^ Research Institute, Casa Colina Hospital and Centers for Healthcare, Pomona, CA, United States; ^6^ Neuromotor Recovery and Rehabilitation Center, David Geffen School of Medicine, University of California, Los Angeles, CA, United States; ^7^ Brain Research Institute, University of California, Los Angeles, CA, United States

**Keywords:** spinal cord injury (SCI), machine learning, acute rehabilitation, computational modelling, functional outcomes

## Abstract

**Introduction:**

Spinal cord injury (SCI) presents a significant burden to patients, families, and the healthcare system. The ability to accurately predict functional outcomes for SCI patients is essential for optimizing rehabilitation strategies, guiding patient and family decision making, and improving patient care.

**Methods:**

We conducted a retrospective analysis of 589 SCI patients admitted to a single acute rehabilitation facility and used the dataset to train advanced machine learning algorithms to predict patients' rehabilitation outcomes. The primary outcome was the Functional Independence Measure (FIM) score at discharge, reflecting the level of independence achieved by patients after comprehensive inpatient rehabilitation.

**Results:**

Tree-based algorithms, particularly Random Forest (RF) and XGBoost, significantly outperformed traditional statistical models and Generalized Linear Models (GLMs) in predicting discharge FIM scores. The RF model exhibited the highest predictive accuracy, with an R-squared value of 0.90 and a Mean Squared Error (MSE) of 0.29 on the training dataset, while achieving 0.52 R-squared and 1.37 MSE on the test dataset. The XGBoost model also demonstrated strong performance, with an R-squared value of 0.74 and an MSE of 0.75 on the training dataset, and 0.51 R-squared with 1.39 MSE on the test dataset. Our analysis identified key predictors of rehabilitation outcomes, including the initial FIM scores and specific demographic factors such as level of injury and prehospital living settings. The study also highlighted the superior ability of tree-based models to capture the complex, non-linear relationships between variables that impact recovery in SCI patients.

**Discussion:**

This research underscores the potential of machine learning models to enhance the accuracy of outcome predictions in SCI rehabilitation. The findings support the integration of these advanced predictive tools in clinical settings to better guide decision making for patients and families, tailor rehabilitation plans, allocate resources efficiently, and ultimately improve patient outcomes.

## Introduction

Spinal cord injury (SCI) is a devastating condition that results in significant physical, psychological, and social disability that impacts not only patients but also their families and the healthcare system. SCI is a highly heterogenous disease, with variability in mechanism of injury, levels of injury, and severity of injury among other factors. The complexity of SCI necessitates a multifaceted approach to both acute treatment and rehabilitation, involving multimodal clinical care and various clinical and functional assessments to track patient progress and outcomes. It is estimated that approximately 273,000 people in the U.S. suffer from SCI with 12,000 new cases each year, leading to significant healthcare utilization and long-term disability (1, 2). Patients often face a spectrum of physical and mental health issues, including chronic pain, spasticity, autonomic dysreflexia, cardiovascular disease, pressure ulcers, urinary tract infections, respiratory complications, and psychological disorders, which severely impact their quality of life and independence (3, 4). These issues also contribute to increased healthcare utilization, including clinic and ED visits to address the sequelae of SCI. For these reasons, SCI patients require extensive social and financial support to manage their condition and associated comorbidities (2).

Predicting functional outcomes for SCI patients is crucial for guiding patient, family, and clinician decision-making in the acute setting as well as for optimizing rehabilitation strategies. Traditional outcome measures, such as the American Spinal Injury Association (ASIA) Impairment Scale and the Functional Independence Measure (FIM), provide a standardized approach to assessing patient status but fall short in predicting long-term outcomes with the desired granularity and accuracy. The Functional Independence Measure is a validated score that includes 18 items divided into motor and cognitive domains, each scored on a scale from 1 (total assistance) to 7 (complete independence). The total FIM score ranges from 18 to 126, with higher scores indicating greater independence (5). FIM has been widely used in various settings and populations, including SCI patients. Studies have demonstrated its reliability and validity in assessing functional outcomes. For instance, Saltychev et al. highlighted the high internal consistency of FIM (5). Similarly, Barbetta et al. demonstrated its validity in SCI, showing a correlation between FIM scores and level of injury (6). Importantly, prior work has suggested improvements in function obtained after SCI are likely to be permanent. Osterthun et al. examined the long-term functional independence of individuals with motor complete SCI using the SCIM III showing that functional gains are often long-lasting (7).

The heterogenous nature of SCI with wide variability in injury mechanisms, levels, and severity as well as diverse patient demographics and clinical characteristics results in non-linear recovery trajectories that are challenging to predict using traditional statistical models. Machine Learning (ML) offers a powerful alternative, as its algorithms are designed to process vast amounts of high-dimensional data and identify complex patterns and non-linear relationships without pre-specified assumptions (8–17). By leveraging demographic information, injury characteristics, and initial functional assessments, ML can generate personalized predictions that guide clinical decision-making and rehabilitation strategies with greater accuracy than traditional methods (18–21). This capability is essential for moving towards more precise and individualized patient care in SCI rehabilitation.

Previous studies have demonstrated the potential of machine learning in predicting outcomes of various neurological conditions, such as traumatic brain injury, cervical spinal cord injury, and strokes (22–26). In the study by Say et al., machine learning models, particularly tree-based algorithms like Random Forests and XGBoost, were successfully applied to predict improvements in Functional Independence Measure scores in patients with traumatic brain injuries undergoing inpatient rehabilitation (25). The ML models demonstrated high accuracy and outperformed traditional statistical methods, showcasing their potential to enhance personalized patient care and optimize resource allocation in rehabilitation settings. While ML has been applied to SCI populations, most prior studies have focused on predicting neurological recovery following surgery or during acute care. For instance, Shimizu et al. developed ML models to predict motor outcomes after cervical SCI surgery and integrated MRI and clinical data to forecast post-surgical outcomes (9, 14, 27). Meanwhile, inpatient rehabilitation is a cornerstone of SCI recovery, providing structured, multidisciplinary care that maximizes functional independence. Accurate prediction of functional status at discharge is especially valuable in this setting, as it helps clinicians tailor therapy intensity, prioritize interventions, and set appropriate recovery goals. A smaller number of studies have explored ML applications during rehabilitation, but these typically focus on single outcome measures, namely ASIA grade or a specific motor task, and do not capture the full scope of patient independence as assessed by all 18 FIM items (12, 23, 28).

To our knowledge, no prior work has used ML to comprehensively predict discharge outcomes across all FIM domains based on data available at admission to inpatient rehabilitation. Therefore, we aimed to address this knowledge gap by applying advanced ML algorithms to a large, comprehensive SCI rehabilitation dataset. Specifically, we evaluated a diverse suite of machine learning models, each selected for its unique strengths in handling the complex and heterogeneous data characteristic of spinal cord injury rehabilitation. We investigated traditional but powerful regression techniques, including Generalized Linear Models (GLMs) and ordinal regression. The use of ordinal regression is specifically motivated by the nature of the FIM score, which is an ordinal variable where the intervals between values are not uniform. These models are highly interpretable, as their coefficients offer clear insights into the magnitude and direction of each feature's impact on rehabilitation outcomes. To enhance these models and prevent overfitting, we applied regularization techniques such as Lasso (L1), Ridge (L2), and Elastic Net. Lasso regression performs automatic feature selection by forcing the coefficients of less important features to zero, while Ridge regression is effective at handling multicollinearity by shrinking coefficients without eliminating them. Elastic Net combines both L1 and L2 penalties, often providing a balanced and robust solution.

More centrally, we focused on advanced tree-based ensemble algorithms, which the study found to be superior at modeling the complex, non-linear relationships inherent in SCI recovery. Random Forest (RF), which emerged as the top-performing model, operates by constructing a multitude of decision trees and averaging their predictions to reduce variance and protect against overfitting. We also evaluated gradient boosting models like XGBoost and CatBoost, which build trees sequentially, where each new tree is trained to correct the errors of the ones before it. XGBoost is a highly efficient and powerful implementation of this method. CatBoost offers a key advantage with its novel, built-in algorithm for processing categorical data, which avoids the need for extensive manual preprocessing and reduces the risk of overfitting, making it a reliable model for datasets with numerous categorical features.

By systematically comparing these distinct approaches, this study provides several novel contributions specifically for the spinal cord injury rehabilitation. First, we conducted a comprehensive and systematic comparison of eleven distinct models, ranging from regularized linear regressions to advanced tree-based ensembles. Our results demonstrate the superior performance of tree-based models, such as RF and CatBoost, in capturing the complex, non-linear dynamics of functional recovery after SCI, thereby establishing a benchmark for future predictive modeling efforts in this domain. Second, beyond pure prediction, our study performs a detailed feature importance analysis using interpretable GLMs and RF. This allows us to identify and quantify the impact of key clinical and demographic predictors, including initial FIM scores, level of injury, and prehospital living settings, offering actionable insights for clinicians. Finally, we articulate a clear pathway for integrating these predictive tools into clinical practice to enhance patient counseling, tailor rehabilitation plans, and optimize the allocation of healthcare resources, bridging the gap between advanced computational analysis and practical clinical decision-making.

## Materials and methods

### Ethical approval and data acquisition

This study was approved by the Institutional Review Board (IRB) of the University of California, Los Angeles (IRB #15-001380). Due to the retrospective nature of the study, the requirement for informed consent was waived. All data were anonymized to ensure patient confidentiality and privacy.

### Study design and participants

This study undertakes a comprehensive retrospective analysis of a prospectively collected dataset, focusing on all patients with traumatic spinal cord injury admitted to the Casa Colina Acute Rehabilitation Unit in Pomona, CA, USA. The dataset encompasses patient admissions spanning from 2010 to 2015, providing a robust longitudinal perspective on rehabilitation outcomes. A total of 589 patients were included in the study. The target inclusion criteria for this study centered on adult patients who had sustained a spinal cord injury and required inpatient rehabilitation following their initial hospital discharge. Only patients with complete functional outcome data, those with FIM scores recorded at both admission and discharge, were included in the final dataset used for analysis. This criterion ensured the reliability and completeness of outcome assessments across all individuals analyzed. To ensure the accuracy and relevance of the findings, specific exclusion criteria were applied. Pediatric patients, defined as individuals under the age of 18, were excluded due to the different nature of pediatric SCI and the distinct rehabilitation protocols typically employed for younger patients. Pregnant patients were also excluded to avoid confounding variables related to pregnancy that could impact rehabilitation outcomes and to adhere to ethical considerations regarding the inclusion of vulnerable populations. Additionally, patients who passed away during rehabilitation were excluded, as mortality prediction falls outside the scope of this study.

### Data collection

Demographic and clinical data were collected from electronic medical records (EMRs). Collected variables included age, sex, race, level of injury (cervical, thoracic, lumbar, sacral), ASIA Impairment Scale (AIS) grade, duration of injury, comorbid conditions, and length of stay (LOS) in the rehabilitation program. FIM scores were documented for each patient. These scores were recorded at two critical time points: at the time of admission to the rehabilitation facility and at the time of discharge.

### FIM scores

The FIM instrument encompasses 18 items across motor and cognitive domains to assess patients' abilities to perform daily activities. The 18 items are: eating, grooming, bathing, dressing the upper body, dressing the lower body, toileting, bladder control, bowel control, bed transfer, toilet transfer, tub transfer, walking/wheelchair use, stair navigation, comprehension, expression, social interaction, problem-solving, and memory. Each item is scored individually as an integer value ranging from 1 to 7, where 7 represents “complete independence,” and 1 indicates “total dependence.” FIM scores were treated as ordinal data rather than points on a continuous scale.

Upon admission, FIM scores served as a baseline measure to capture the severity of the impairment and the initial functional capabilities. At discharge, FIM scores were measured again to assess the degree of improvement achieved during the rehabilitation stay. The difference between admission and discharge FIM scores provided a quantifiable measure that reflected the effectiveness of the rehabilitation interventions.

### Predictive parameters

In our effort to develop prediction models, we utilized a comprehensive dataset incorporating 28 numerical features and 10 categorical features. This robust dataset was designed to accurately predict rehabilitation outcomes, leveraging a blend of quantitative and qualitative data to enhance model performance.

During the data preprocessing phase, values for certain features were absent. To address missing values in numerical features, we employed mean imputation due to its simplicity and minimal risk of introducing information leakage or artificial variance. This technique involves calculating the mean of the observed values for each numerical feature and substituting this mean for any missing entries. By doing so, we ensured that the imputed values reflected the central tendency of the data without introducing significant bias. Mean imputation is particularly beneficial as it leverages available data to fill in gaps, preserving the dataset's integrity and variability and is a well-established method for addressing missing data. More sophisticated techniques, such as multiple imputation or k-nearest neighbors (kNN), often use inter-variable correlations to predict missing values. If not perfectly nested within a cross-validation framework, and in lack of sufficient training data, these methods can lead to data leakage from the validation set to the training set, resulting in overly optimistic performance estimates and a model that generalizes poorly. Our approach prioritized the avoidance of such algorithmic bias that could jeopardize the generalization and applicability of our conclusions. By using the mean—a single measure of central tendency calculated solely from the training data within each validation fold—we ensured that no artificial relationships are introduced into the dataset for the model to learn from, thereby maintaining the integrity of the model validation process.

For categorical features, absent entries were treated by the model as a distinct category. Subsequently, we applied a one-hot encoding technique to transform categorical features into a binary format suitable for machine learning algorithms. One-hot encoding converts each categorical feature into a set of binary (0 or 1) variables, with each binary variable representing the presence or absence of a particular category. This transformation expanded the dataset to include 85 predictive variables, significantly increasing its dimensionality and ensuring that all categorical information was comprehensively captured.

### Machine learning model development

Our objective was to identify optimal machine learning models that minimize prediction error for rehabilitation outcomes, specifically focusing on FIM scores at discharge. We used both R (R Studio Version 2023.03.1 + 446) and Python (version 3.10.9) to evaluate the performance of eleven different models. These included three ordinal regression models [Lasso ordinal regression, Elastic-Net (EN) regression, and Ridge regression], three generalized linear models (Lasso, EN, and Ridge GLM), four tree-based methods (XGBoost, Random Forest, CatBoost, and LightGBM), and a baseline approximation. A baseline model, in which the admission FIM scores were considered as the predicted discharge scores, served as a control.

To perform ordinal regression, we utilized the “ordinalNet” package (v 2.12) in R. Ordinal regression is suited for predicting ordinal variables, which are variables with a clear ordering but unknown intervals between values. This type of regression serves as an intermediate approach between standard regression and classification problems. To mitigate overfitting, we applied three regularization techniques: Lasso, Elastic Net (EN), and Ridge regression. These techniques introduce a penalty term to the loss function to control the complexity of the model. Lasso regression employs a penalty proportional to the L1 norm of the parameter vector, Ridge regression uses the L2 norm, and Elastic Net combines both L1 and L2 penalties. Similar regularization methods were applied to GLMs using the “glmnet” package (v 4.1.7) in R.

### Hyperparameter optimization of tree-based models

To enhance the performance of our tree-based models, we conducted hyperparameter optimization using a 10-fold cross-validation method. This process involves fine-tuning the following hyperparameters:

• XGBoost: We optimized seven hyperparameters—max_depth, nrounds, eta, gamma, colsample_bytree, min_child_weight, and subsample.
• Random Forest: Four hyperparameters were tuned—mtry, maxmode, ntree, and Nodesize.
• CatBoost: Three hyperparameters were optimized—iterations, learning_rate, and depth.
• LightGBM: Three hyperparameters were adjusted—learning_rate, max_depth, and num_leaves.

The optimal parameter sets, determined based on test set performances, were used to train the final models.

### Model validation

To prevent overfitting and ensure robust model performance, we employed a standard five-fold cross-validation technique during model training. This method involves randomly dividing the dataset into five equal-sized folds. In each iteration of training, one fold is used for validation while the remaining four folds are used for training. This process is repeated five times, resulting in five distinct models. The model with the highest accuracy from these five iterations is selected as the final model.

### Evaluation of model performance

We assessed the performance of our models in predicting FIM scores at discharge using two key metrics: Mean Squared Error (MSE) and R-squared (R^2^) values. MSE is calculated as the average squared difference between the predicted values and the actual values, with lower MSE indicating better predictive accuracy. R^2^ measures the proportion of variance in the dependent variable that is predictable from the independent variables, with higher R^2^ values indicating better model fit. This means that high-performing models exhibit low MSE and high R^2^ values.

### Feature importance

In addition to evaluating overall model performance, we examined the importance of individual features within the GLM models. GLMs provide selected features along with their respective coefficients, which indicate the strength and direction of the relationship between each feature and the predicted outcome. By analyzing the frequency with which each feature is included in the models and the magnitude of its coefficient, we can identify the most influential features in predicting rehabilitation outcomes.

### Software and tools

All data preprocessing, model development, and statistical analyses were conducted using Python (version 3.10.9) and R (R Studio Version 2023.03.1 + 446). Key libraries included scikit-learn for machine learning models and pandas for data manipulation, while data visualization was performed using the matplotlib and seaborn libraries.

## Results

### Study participants

Table 1 provides a breakdown of demographic and clinical data points of patients. Overall, 589 SCI patients were included in the study, and 369 patients were male (63%). The average age was 58.5 years. Comorbidities, which can significantly influence functional ability after SCI, were assessed using ICD codes (29). Diabetes mellitus was present in 88 participants (15%), and coronary artery disease in 22 participants (4%), with other comorbidities (e.g., dementia, metastatic cancer) absent in this cohort. For pre-admission living situation, the vast majority, 558 patients (95%), resided at home, with others living in settings such as board and care, transitional living, and skilled nursing facilities. Prior to admission, 479 (81%) lived with family or relatives, 60 (10%) lived alone, and the remainder lived with friends, attendants, or others. During admission, most patients, 458 (78%), had a regular diet, 103 (17%) required modified food consistency or supervision, and 25 (4.2%) needed tube or parenteral feeding. For ambulation, measured as the ability to walk or requirement of a wheelchair, 311 patients (53%) were categorized as Walk (W), 257 (44%) as Wheelchair (C), and 21 (4%) as Both (B). Comprehension is categorized as Auditory (A), Visual (V), or Both (B) with 344 patients (58%) as B (i.e., comprehending both auditory and visual cues), 227 (39%) as A, and 18 (3%) as V. Similar categories are used for expression, with 314 (53%) as B (i.e., using both vocal and nonvocal expressions), 273 (46%) as V, and 2 (0.3%) as Nonvocal (N). The average LOS of patients in rehabilitation facilities is 22.78 days.

**Table 1:** Baseline categorical characteristics of the study population, *N* = 589.

Features		*N* (%)
Gender	Female	220 (37%)
	Male	369 (63%)
Diabetes mellitus	True	88 (15%)
	False	501 (85%)
Coronary artery disease	True	22 (4%)
	False	567 (96%)
PreHospitalLivingSetting	Home	558 (95%)
	Board and care	3 (0.51%)
	Transitional living	4 (0.68%)
	Intermediate care	1 (0.17%)
	Skilled nursing facility	1 (0.17%)
	Rehabilitation facility	1 (0.17%)
	Assisted living residence	4 (0.68%)
	Other	4 (0.68%)
	Unknown	13 (2.2%)
PreHospitalLivingWith	Alone	60 (10%)
	Family/Relatives	479 (81%)
	Friends	12 (2.0%)
	Attendant	4 (0.68%)
	Other	3 (0.51%)
	Unknown	31 (5.3%)
Admit-Swallowing Status	Tube/Parenteral Feeding	25 (4.2%)
	Modified Food Consistency/Supervision	103 (17%)
	Regular Food	458 (78%)
	Unknown	3 (0.51%)
Admit-FIMWalkWheelchairMeasured	B	21 (4%)
	C	257 (44%)
	W	311 (53%)
Admit-FIMComprehensionMeasured	A	227 (39%)
	B	344 (58%)
	V	18 (3%)
Admit-FIMExpressionMeasured	B	314 (53%)
	N	2 (0.3%)
	V	273 (46%)
Complications	Intraspinal abscess	12 (2.0%)
	Late effect of intracranial abscess or pyogenic infection	3 (0.51%)

### Functional assessments and measures

Table 2 provides a summary of various functional assessments and measures for the patient population, captured at the time of admission to the rehabilitation facilities. The table reports average scores with standard deviations for activities of daily living (ADLs). In addition to the scores for FIM items (AdmitFIM), it also reports the level of assistance required for bladder and bowel management, frequency of accidents, and modifications needed for walking and wheelchair use (AdmitFnMod). The data indicate a quantitative summary of the patient's functional status upon admission, which is critical for planning rehabilitation and care interventions.

**Table 2:** Baseline functional numerical characteristics of the study population, *N* = 589.

Features	Mean (sd)
LOS	22.78 (16.5)
AGE	58.47 (18.84)
AdmitFIMEating	4.41 (1.96)
AdmitFIMGrooming	3.95 (1.77)
AdmitFIMBathing	2.29 (1.26)
AdmitFIMDressingUpper	3.1 (1.68)
AdmitFIMDressingLower	1.85 (1.19)
AdmitFIMToileting	1.55 (1.05)
AdmitFIMBladderCtrl	2.16 (1.87)
AdmitFIMBowelCtrl	3.04 (2.26)
AdmitFIMBedTransfer	2.03 (1.22)
AdmitFIMToiletTransfer	2.01 (1.36)
AdmitFIMTubTransfer	1.18 (1.63)
AdmitFIMWalkWheelchair	1.88 (1.63)
AdmitFIMStairs	0.49 (0.97)
AdmitFIMComprehension	5.9 (1.22)
AdmitFIMExpression	5.9 (1.32)
AdmitFIMSocialInteraction	5.98 (1.28)
AdmitFIMProblemSolving	5.4 (1.45)
AdmitFIMMemory	5.48 (1.46)
AdmitFnModBladderLvlAssist	2.17 (1.87)
AdmitFnModBladderFreqAccidents	5.8 (0.78)
AdmitFnModBowelLvlAssist	3.04 (2.26)
AdmitFnModBowelFreqAccidents	5.81 (0.71)
AdmitFnModDistWalked	0.93 (0.96)
AdmitFnModDistWheelchair	1.58 (1.01)
AdmitFnModWalk	1.04 (1.22)
AdmitFnModWheelchair	2.07 (1.79)

**Figure 1:**
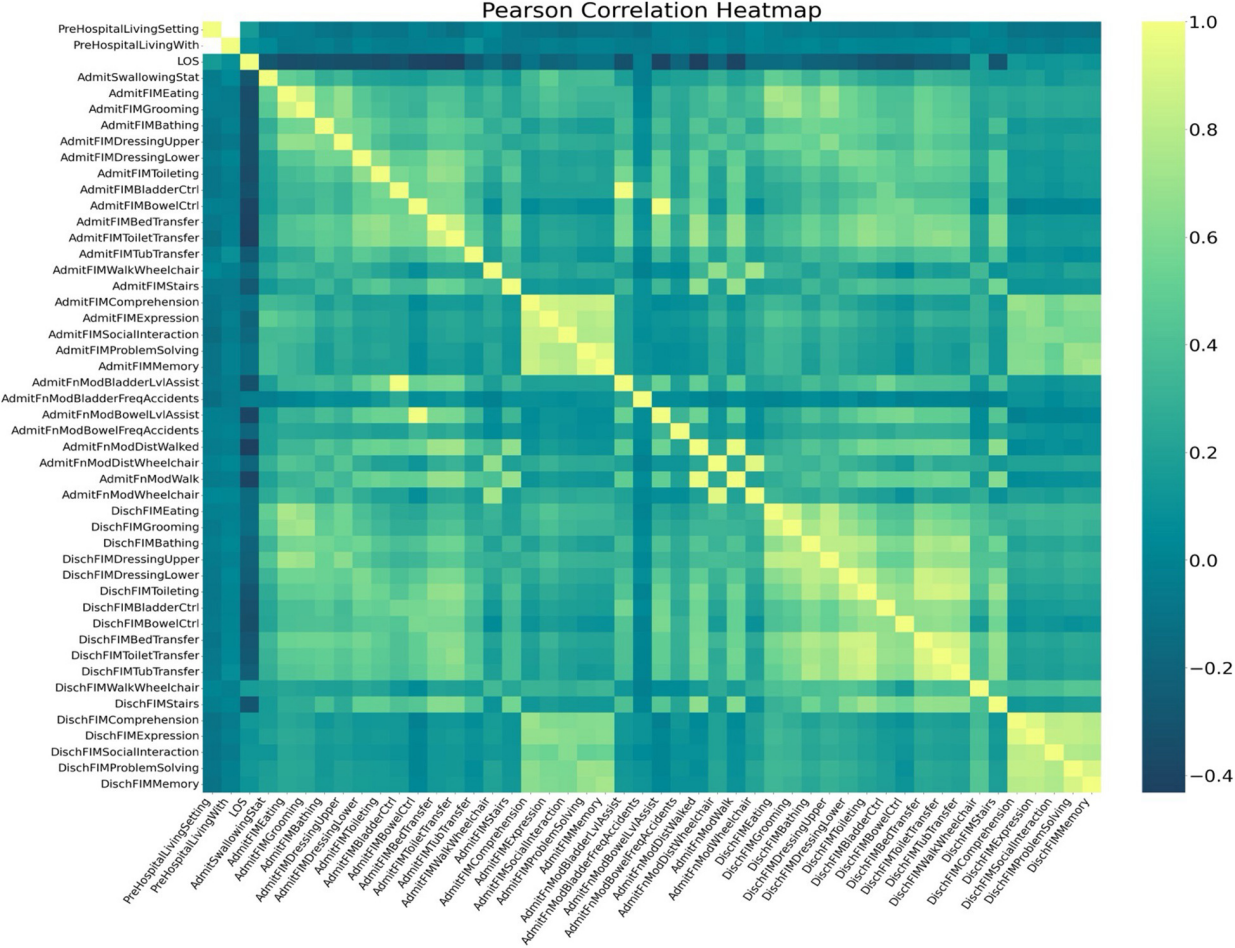
Pearson correlation map showing the correlations between all variables. A positive correlation coefficient (yellow) indicates a positive linear relationship, while a negative correlation coefficient (dark blue) indicates a negative linear correlation.

Figure 1 displays a Pearson correlation heatmap between variables related to patient functional status at admission (AdmitFIM) and discharge (DischFIM) as well as other potential factors such as LOS. The variables on both axes are the same, allowing for a symmetrical comparison of correlations. The color gradient ranges from yellow to dark green, with yellow indicating a strong positive correlation (1.0) and dark green indicating a moderately negative correlation (−0.4). Gradients between these colors indicate the respective strength of correlation. FIM scores associated with physical activity (such as Bladder Control, Toileting, Tub Transfer, etc.) positively correlate with one another, while FIM scores associated with social activity (such as Comprehension, Expression, Social Interaction, etc.) correlate very strongly with each other. Furthermore, there appear to be very few strong negative correlations, as evidenced by the lack of dark green squares in the heatmap. Notably, LOS of the cohort shows strong negative correlations with all other variables, indicating connections between lower FIM scores and longer stays in the rehabilitation facilities. As would be expected, the admission FIM score for a given domain (i.e., grooming) tended to correlate strongly with the discharge FIM score for the same domain.

**Figure 2:**
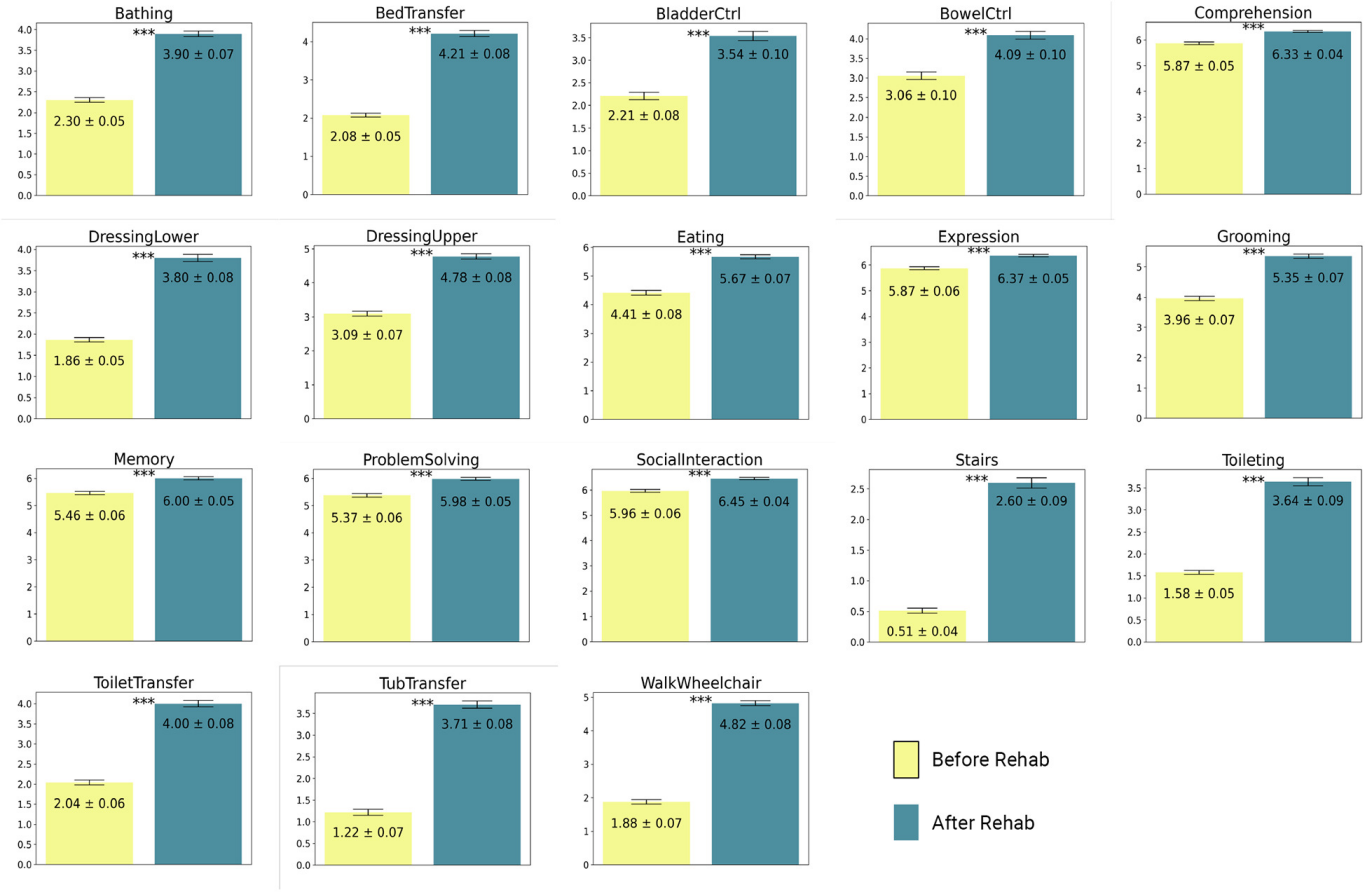
T-test bar plots of FIM scores with pre-rehab scores shown in yellow and post-rehab scores shown in blue. All metrics showed a statistically significant improvement after rehab. ***p < 0.001.

Figure 2 presents bar graphs that detail the mean scores of various FIM items before and after rehabilitation. The yellow bars represent the FIM scores prior to rehabilitation, while the green bars indicate the scores after rehabilitation. Each graph is labeled with the specific FIM task being measured. Error bars are included to represent the standard deviation, providing a visual depiction of the variability within each dataset and underscoring the reliability of the measurements. This figure shows significant improvements in functional independence following rehabilitation. The scores for dressing, eating, toileting, bathing, grooming, bed transfer, tub transfer, bladder control, bowel control, stairs, cognitive comprehension, expression, memory, problem solving, and social interaction, all show significant improvements. The results of paired t-tests comparing admission and discharge FIM scores reveal statistically significant improvements across all 18 FIM scores (Figure 2; *p* < 0.001), providing strong evidence of the positive impact of rehabilitation on patient outcomes. These findings highlight the efficacy of rehabilitation programs in enhancing patients' functional independence, consistent with our current understanding of the importance of rehabilitation after SCI.

### Unsupervised classification using principal component analysis (PCA)

To explore the underlying structure of the data and identify patterns, we performed a Principal Component Analysis (PCA) using the FactoMineR package (v 2.9). The PCA was conducted on the dataset comprising the eighteen dependent variables. The discharge FIM scores ranged from 1 to 7, with higher scores represented by lighter colors and lower scores by darker colors. The eigenvalues indicate the amount of variance captured by each principal component, with higher eigenvalues signifying greater explained variance. We analyzed the contributions of the variables to the first and second principal components and highlighted the top 10 contributing variables for each component. The overall contributions of the variables were visualized, the arrows representing independent variables are color-coded by their contribution values, computed based on the squared cosine (cos^2^) values. The cos^2^ values indicate the quality of representation of the variable on the principal component.

**Figure 3:**
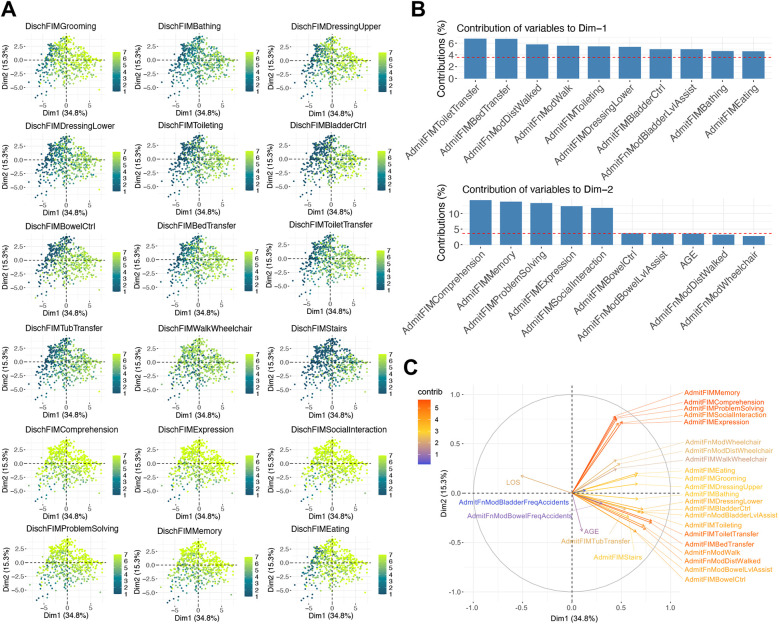
PCA scatter plots and variable contributions. The scatter plots **(A)** visualize individuals color-coded by their discharge FIM scores. Higher scores are shown with more yellow colors. The bar plots **(B)** display the contributions of variables to the first and second principal components, and the radar plot **(C)** illustrates the overall variable contributions with arrows color-coded by their contribution values.

Figure 3 presents the PCA scatter plots and variable contributions. The scatter plots (Figure 3A) display individuals color-coded by their discharge FIM scores, revealing clusters of individuals with similar functional independence levels. The bar plots (Figure 3B) show the contributions of the variables to the first and second principal components, with variables such as AdmitFIMWalkWheelchair, AdmitFIMBathing, and AdmitFIMToiletTransfer exhibiting high contributions. The radar plot (Figure 3C) further elucidates the overall variable contributions, emphasizing the significant role of specific functional measures at admission in explaining the variance in rehabilitation outcomes. The PCA results indicate that certain admission FIM scores, particularly those related to mobility and self-care, are critical in defining the principal components, thus influencing the overall rehabilitation outcomes.

### Generalized linear models optimization

We performed hyperparameter tuning for GLMs; the principal tool employed for this purpose was the “glmnet” function, version 4.1–7. This tool, as outlined by the protocols at https://glmnet.stanford.edu/reference/cv.glmnet.html, facilitated the robust cross-validation and regularization of our models. We adopted a 5-fold validation strategy to fine-tune the regularization parameter, lambda, effectively varying it across a logarithmic scale from 10^2^ down to 10^−3^.

The choice of lambda depended on achieving the minimal mean cross-validated error (cvm), a predefined metric provided by the package. This methodical approach ensured that each model configuration was optimized for both accuracy and complexity, mitigating the risk of overfitting while enhancing predictive performance. The fine-tuning of lambda within the designated range allowed us to explore a spectrum of model behaviors, from highly regularized to more flexible fits, thereby identifying the setting that optimally balanced error minimization and model complexity.

### Tree-based models optimization

We employed a grid search approach to systematically optimize the hyperparameters for four tree-based models: Random Forest (RF), XGBoost, LightGBM, and CatBoost. This method was chosen to ensure the highest possible model accuracy while preventing overfitting by exploring various combinations of parameter values and selecting those that yielded the best performance on the test set. For the Random Forest model, hyperparameter tuning focused on four key parameters. The number of variables randomly sampled as candidates at each split was varied between 30, 40, and 50. The maximum number of terminal nodes per tree was explored within the range of 5–25. The number of trees in the forest was adjusted from 50 to 1,000 to balance model robustness and computational efficiency. Finally, the minimum size of terminal nodes was tested with values of 5, 10, 15, 100, and 500, which influenced the granularity and detail of the resulting trees.

In the case of XGBoost, seven parameters were tuned to enhance both tree complexity and regularization. The maximum depth of a tree was evaluated at depths of 2, 3, 5, and 7. The number of boosting rounds was varied from 50 to 500 to optimize the balance between training duration and performance. The learning rate was adjusted across 0.01, 0.1, and 1 to control the step size at each iteration. The regularization parameter was explored with values of 0, 1, 5, and 10 to manage overfitting. Additionally, “colsample_bytree,” which controls the number of features supplied to a tree, was tested with fractions of 0.5, 0.75, and 1.0. The minimum sum of instance weight needed in a child node was tuned across 1, 5, and 10 to ensure nodes had enough data points. Lastly, the subsample ratio of the training instances was varied at 0.5, 0.6, and 1 to prevent overfitting by introducing randomness.

For LightGBM, three primary parameters were tuned to optimize performance. The number of leaves in one tree was tested at values of 5, 50, 100, and 500, which directly impacted the complexity of the model. The learning rate was adjusted across 0.0001, 0.001, and 0.01 to fine-tune the shrinkage rate during training. The maximum depth of a tree was evaluated with values of 2, 3, 5, and 7, providing control over the model's complexity and depth. For CatBoost, three parameters were subject to optimization. The number of boosting iterations was varied between 100, 500, and 1,000 to balance the trade-off between training time and model accuracy. The learning rate was tested with values of 0.0001, 0.001, and 0.01 to control the rate of updates to the model. Finally, the depth of the trees was tuned with values of 2, 3, 5, and 7 to manage the model's capacity to learn from the data.

By employing a grid search methodology, we were able to systematically evaluate and identify the optimal hyperparameters for each model, thus enhancing their predictive accuracy and robustness against overfitting. Table 3 shows the results of hyperparameter tuning for four tree-based models. The results displayed in Table 3 are generated by averaging the five sets produced by five-fold validations. The columns display results for eighteen dependent variables, and the rows present the optimal parameters for different models.

**Table 3:** Optimal parameters of tree-based models obtained from hyperparameter tuning.

Model	Parameter	Description	Disch FIM Eating	Disch FIM Grooming	Disch FIM Bathing	Disch FIM Dressing Upper	DischFIM Dressing Lower	DischFIM Toileting	Disch FIM Bladder Ctrl	Disch FIM Bowel Ctrl	Disch FIM Bed Transfer	Disch FIM Toilet Transfer	Disch FIM Tub Transfer	Disch FIM Walk Wheel chair	Disch FIM Stairs	DischFIM Comprehension	Disch FIM Expression	Disch FIM Social Interaction	Disch FIM Problem Solving	Disch FIM Memory
XGBoost	Nrounds	maximal # iteration	70	230	230	320	230	240	290	370	140	440	280	290	120	160	180	180	330	150
	Max_depth	maximal depth of the tree	4.8	4.2	2	3.8	3.4	2.6	3.6	4.8	4	4.8	4.4	2.2	4.4	4.2	4.4	4.2	3.6	3
	Eta	learning rate	0.10	0.10	0.26	0.06	0.10	0.08	0.06	0.08	0.08	0.05	0.08	0.10	0.28	0.10	0.10	0.28	0.06	0.10
	Gamma	regularization	1.2	1.4	2.6	2.2	1.8	2.6	7	10	1.2	2.2	4.2	2.4	5.2	1	1.4	2.4	0.4	2.2
	Colsample_bytree	controls # features supplied	0.5	0.5	0.6	0.6	0.5	0.6	0.7	0.6	0.5	0.6	0.6	0.6	0.7	0.7	0.5	0.7	0.7	0.7
	Min_child_weight	minimal samples in a node	10	10	10	10	10	10	10	10	10	10	10	10	10	10	10	10	10	10
	Subsample	controls # samples supplied	1	1	1	1	1	1	1	1	1	1	1	1	1	1	1	1	1	1
RF	Mtry	variables selected at a split	40	50	30	40	30	50	30	30	40	30	30	40	50	30	40	40	50	50
	Maxnode	maximal # terminal nodes	5	23	7	10	19	20	13	5	23	21	23	18	9	20	6	14	21	16
	Ntree	number of trees	500	500	100	300	500	300	500	300	50	50	1,000	50	100	1,000	500	100	1,000	500
	Nodesize	# samples in terminal nodes	15	5	20	5	15	20	50	5	5	5	5	5	20	5	5	5	5	5
LightGBM	Learning_rate	learning rate	0.01	0.01	0.01	0.01	0.01	0.01	0.01	0.01	0.01	0.01	0.01	0.01	0.01	0.01	0.01	0.01	0.01	0.01
	Max_depth	maximal depth of the tree	7	7	5	5	5	7	3	7	7	5	7	7	5	5	5	5	5	7
	Num_leaves	maximal # terminal nodes	50	50	50	50	50	50	50	50	50	50	50	50	50	5	5	5	50	50
CatBoost	Depth	maximal depth of the tree	5	7	5	5	5	5	3	5	7	5	5	5	3	5	3	5	5	3
	Iterations	maximal # trees created	1,000	1,000	1,000	1,000	1,000	1,000	500	1,000	1,000	1,000	1,000	1,000	1,000	1,000	1,000	1,000	1,000	1,000
	Learning_rate	learning rate	0.01	0.01	0.01	0.01	0.01	0.01	0.01	0.01	0.01	0.01	0.01	0.01	0.01	0.01	0.01	0.01	0.01	0.01

### Model precision

**Figure 4:**
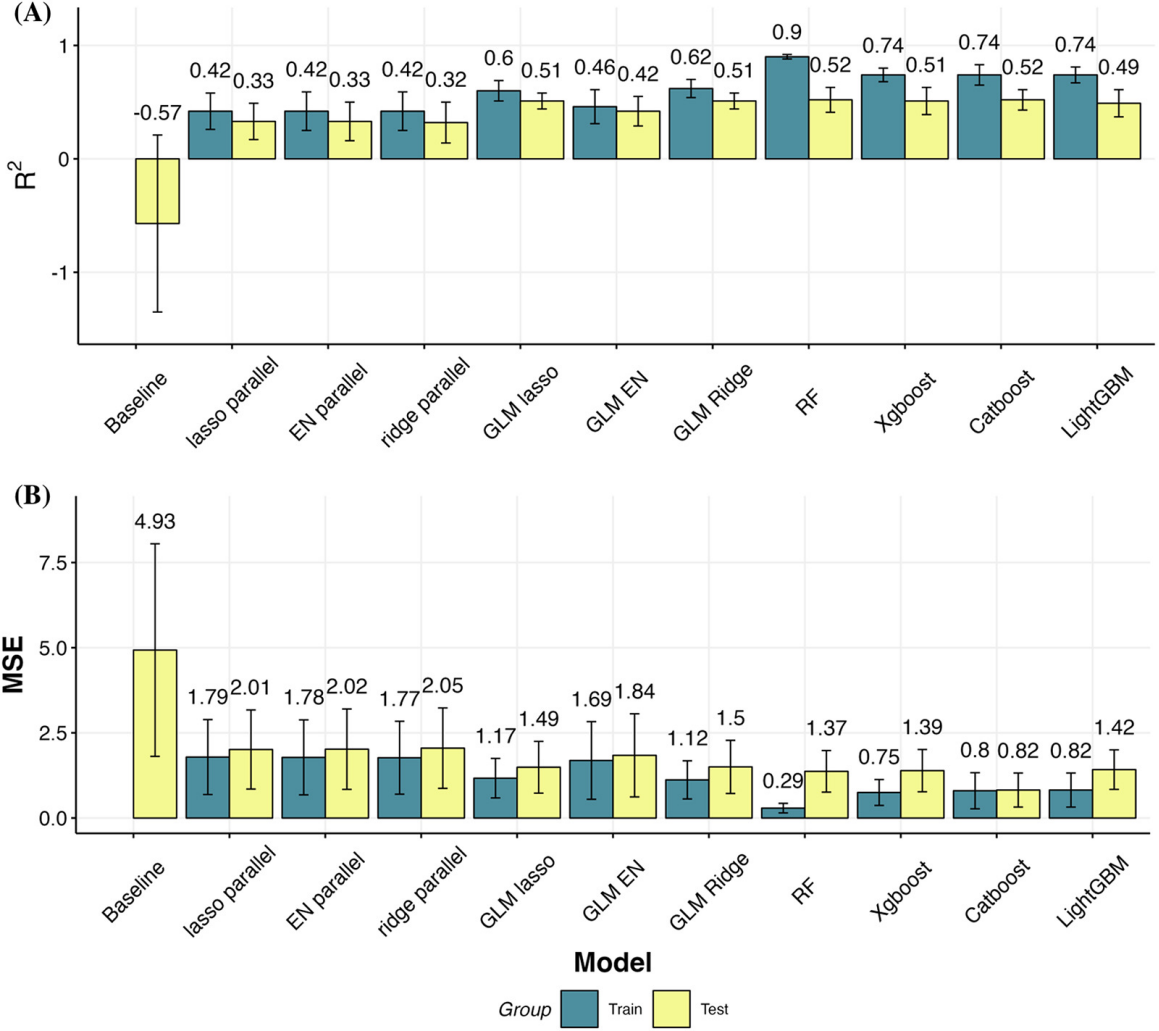
Model performance measured by R-squared and MSE. **(A)** Bar plots with error bars of the R-squared of the train and test sets of models. **(B)** Bar plots with error bars of the Mean Squared Error (MSE) of the train and test sets of models. The highest R-squared value and the lowest MSE value is noted in the RF group, suggestive of a highly accurate model.

Figure 4 presents bar plots illustrating the prediction outcomes of various models. Figure 4A displays the R-squared values, while Figure 4B shows the MSE values. In both subgraphs, blue bars represent the training dataset results, and yellow bars represent the test dataset results. In the training dataset, there are noticeable variations in model performance. The baseline model, which uses the same score at admission as the estimate for discharge, performs poorly, serving as a benchmark for comparison. Ordinal regression models, including lasso, elastic net, and ridge parallel models, show consistent performance with R-squared values around 0.42 and MSE values ranging from 1.77 to 1.79. These models provide a moderate fit to the training data.

Generalized Linear Models demonstrate better performance than ordinal regression models. Specifically, the GLM with lasso regularization achieves an R-squared value of 0.6 and an MSE of 1.17. The ridge regularizations within the GLM framework further improve performance, with the GLM elastic net showing an R-squared value of 0.62 and an MSE of 1.12, indicating better predictive accuracy and lower error. Tree-based models exhibit the strongest performance on the training dataset. Random Forest achieves the highest R-squared value of 0.90 and the lowest MSE of 0.29, indicating exceptional model fit and predictive power. XGBoost and CatBoost also perform well, with R-squared values of 0.74 and MSE values ranging from 0.75 to 0.80, demonstrating robust predictive capabilities with relatively low errors.

Validation on the test sets reveals trends consistent with those observed in the training dataset. The baseline model yields the poorest fit on the test set, with an R-squared value of −0.57 and an MSE of 4.93, indicating a significant mismatch between predicted and actual outcomes. Ordinal regression models show improvement over the baseline, with R-squared values between 0.32 and 0.33 and MSE values between 2.01 and 2.05, suggesting they provide a better but still modest fit. GLM models continue to show better performance on the test set compared to ordinal regression models. The R-squared values for GLM models range from 0.42 to 0.51, and MSE values range from 1.49 to 1.84, indicating more accurate predictions and lower errors. Among the GLM variants, the ridge regularization performs the best, closely followed by the lasso regularization. Tree-based models maintain their superior performance on the test set. The R-squared values for these models range from 0.49 to 0.52, and MSE values are between 0.82 and 1.42, indicating that these models not only fit the training data well but also generalize effectively to new data. CatBoost, in particular, with its tuned hyperparameters, achieves the highest accuracy and minimal overfitting, as indicated by its R-squared value of 0.52 and MSE of 0.82. This suggests that CatBoost is the most reliable model for predicting discharge performances, combining high accuracy with robust generalization capabilities.

**Figure 5:**
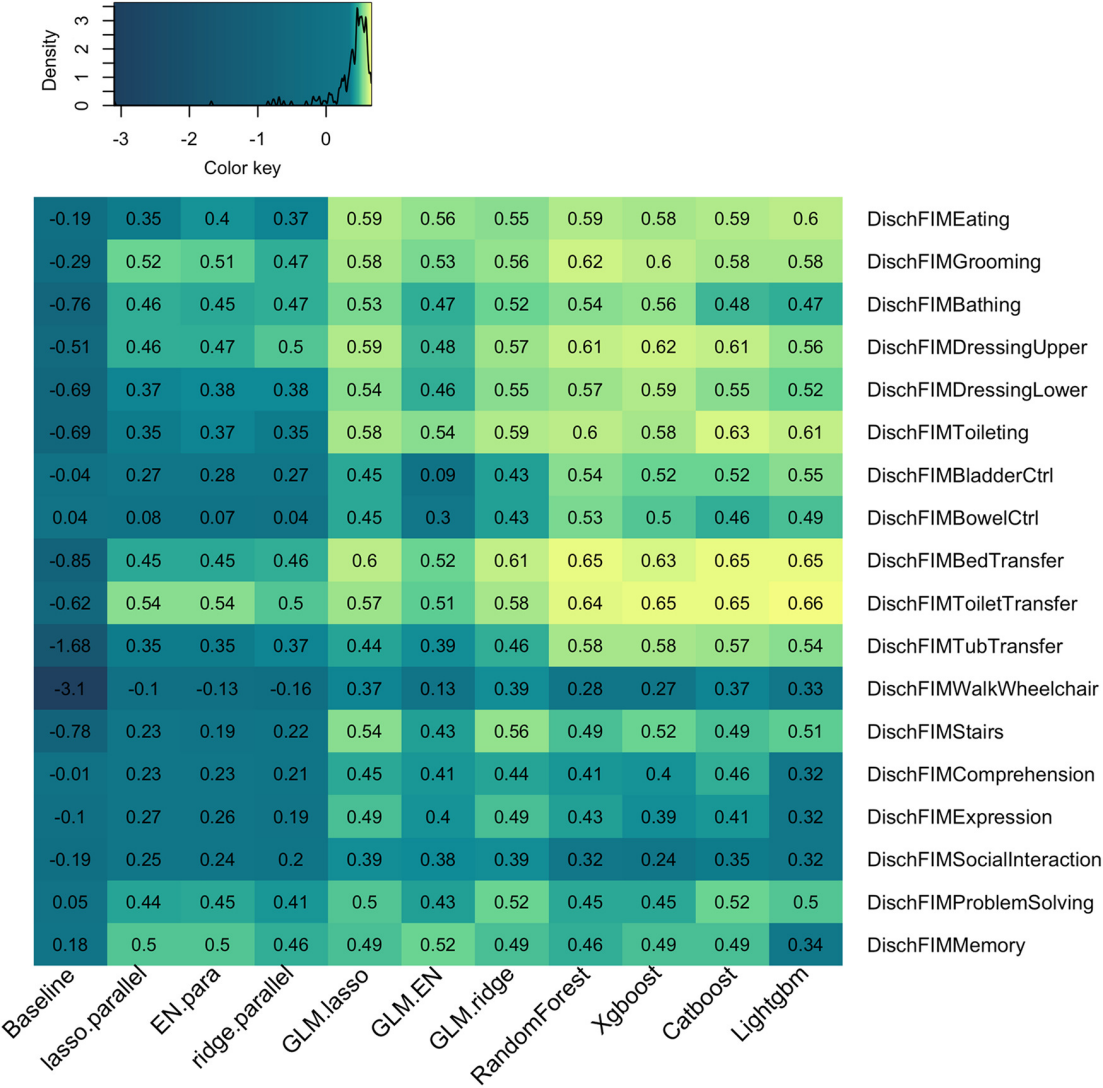
Heatmap of test R-squared for eleven models and eighteen dependent variables. The fill of the heatmap represents the test R-squared value of the given model and dependent variable. The density map on the upper left showed that R-squared values skewed to the left and mostly clustered between 0 and 1. Higher values (shown in yellow) represent higher R-squared values and by extension better model performance.

Figure 5 presents a detailed comparison of model performance across various dependent variables, measured by the R-squared values on the test set. Each cell in the heatmap is color-coded to represent the R-squared value for the corresponding model-variable pair, with lighter colors indicating higher R-squared values. The heatmap reveals that different models fit differently across the various dependent variables. For instance, all models tend to perform better in predicting discharge toilet transfer (DischFIMToiletTransfer) compared to other variables. Conversely, models generally perform poorly in predicting discharge Walk/Wheelchair status (DischFIMWalkWheelchair) and discharge social interaction scores (DischFIMSocialInteraction). When comparing model types, tree-based models and GLMs tend to outperform ordinal regression models across most dependent variables. Specifically, tree-based models exhibit higher R-squared values for variables such as discharge bladder control (DischFIMBladderCtrl), discharge bowel control (DischFIMBowelCtrl), discharge bed transfer (DischFIMBedTransfer), discharge toilet transfer (DischFIMToiletTransfer), and discharge tub transfer (DischFIMTubTransfer). This indicates that tree-based models are more effective in capturing the complexities and interactions within the data, leading to better predictive performance for these variables.

### Importance of clinical features

**Figure 6:**
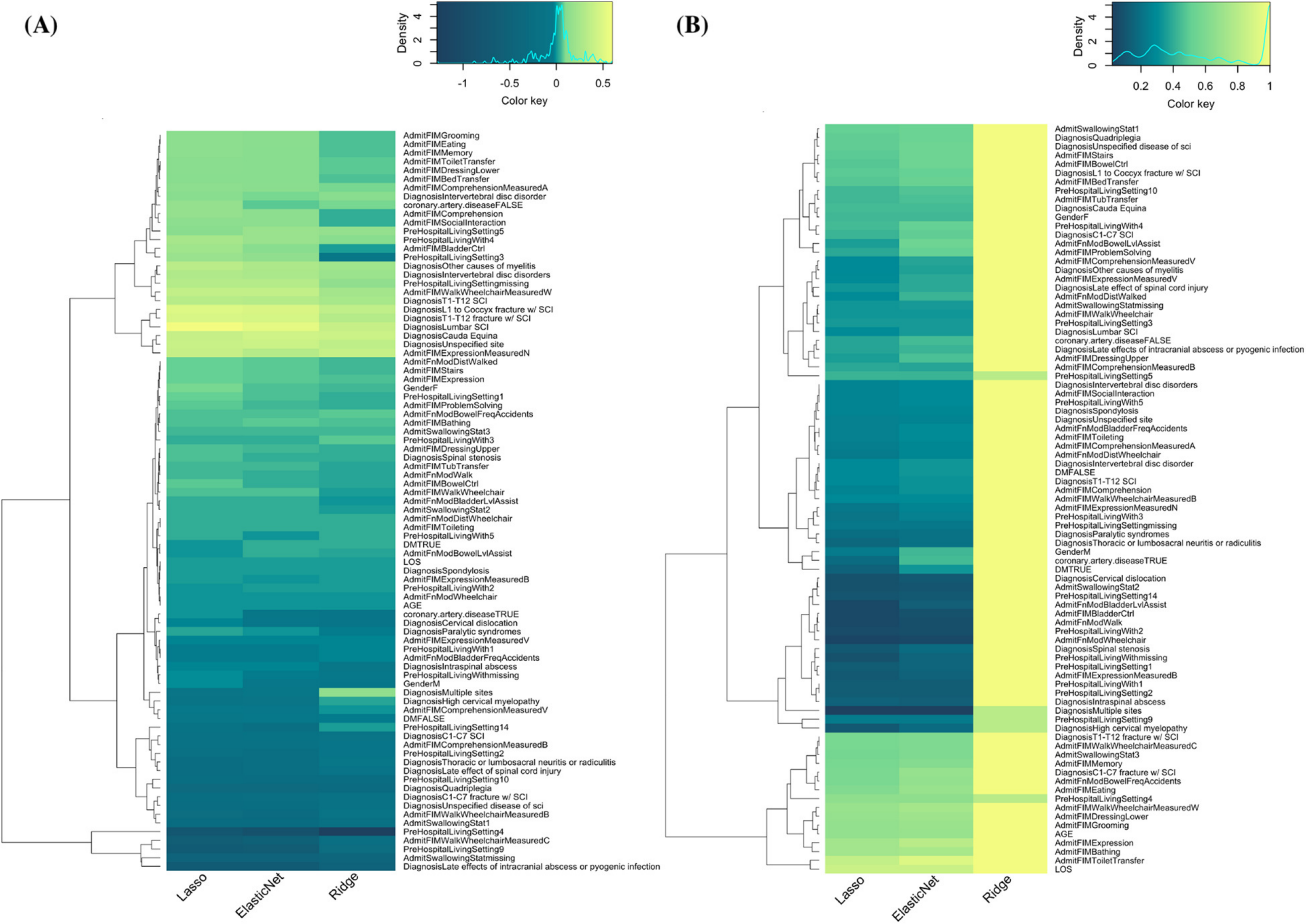
Feature identification using GLM models and dendrogram algorithm. **(A)** Coefficient of independent variables in three GLM models ranked by dendrogram algorithm. **(B)** Frequencies of coefficients being chosen (non-zero) in the three GLM models. On a scale of 0–1, 1 (bright yellow) indicates the variable is selected 100% of the time (*n* = 90).

According to the frequencies of feature inclusion (non-zero coefficients) and their values in GLM models, important features for predicting rehabilitation outcomes are identified. Figure 6 displays the coefficients and frequencies of inclusion in GLM models. In both sub-figures A and B, the *x*-axis represents the three GLM models—Lasso, Elastic Net (EN), and Ridge—while the *y*-axis lists all the independent features.

**Figure 7:**
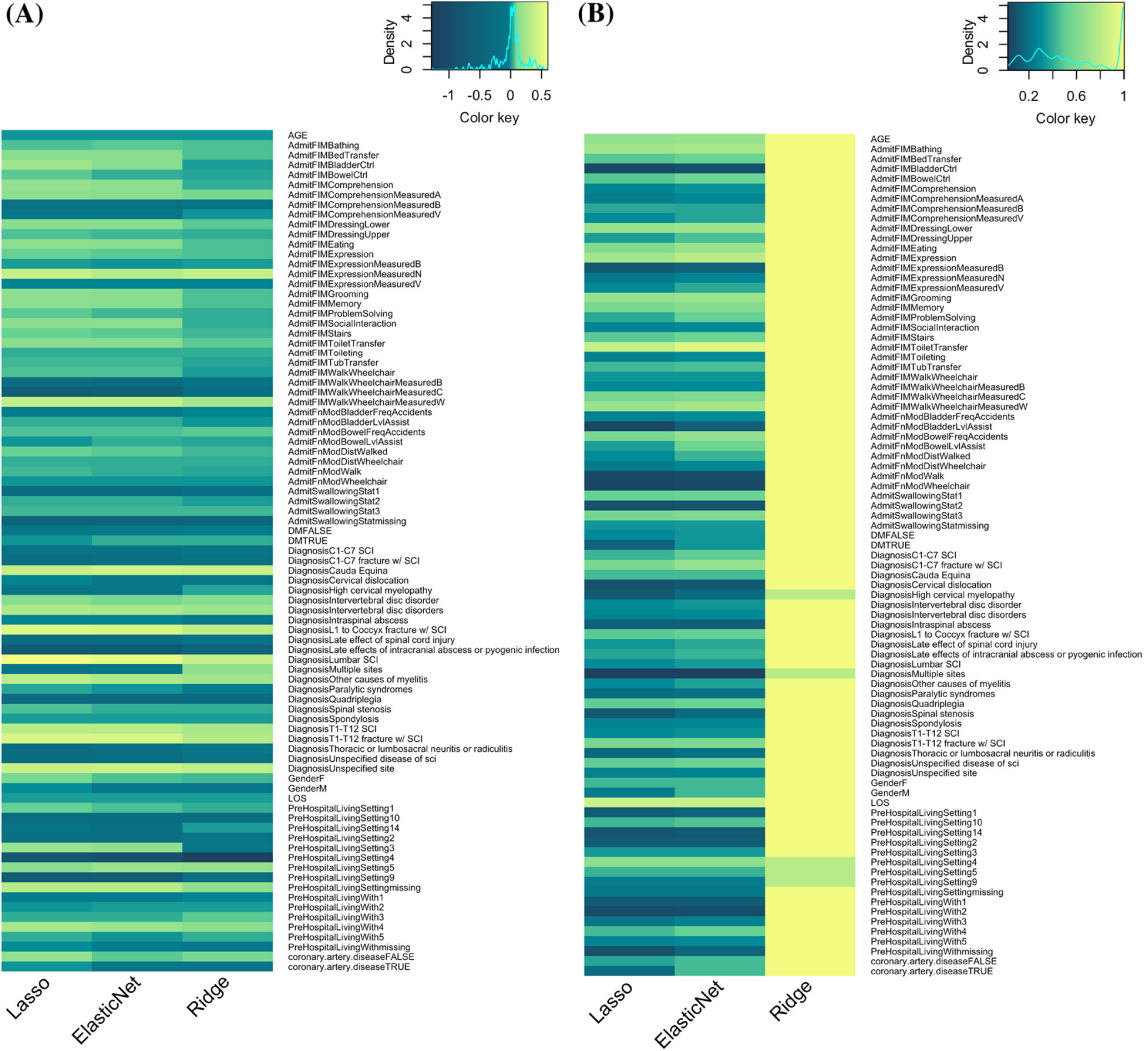
Feature identification using GLM models organized by variable categories. **(A)** Coefficients of independent variables in relation to rehab outcomes, with yellow representing positive outcome predictions and dark green representing negative predictions. Range = −1.28 to 0.6. **(B)** Frequencies of coefficients being chosen (non-zero) in the three GLM models. On a scale of 0–1, 1 (bright yellow) indicates the variable is selected 100% of the time (*n* = 90).

In Figure 6A, the non-zero coefficients extracted from the GLM models are shown. The color scale indicates the magnitude and direction of the coefficients, with yellow representing more positive coefficients and dark green indicating more negative coefficients. From the density map displayed above the heatmap, we observed a normal distribution of coefficient values around 0, depicted by the light green color. The absolute value of a coefficient indicates the impact of the corresponding feature on the overall prediction: a higher absolute value signifies a stronger impact. For visualization and analysis purposes, the order of independent variables is clustered hierarchically, as shown by the dendrogram on the left. This hierarchical clustering uses Euclidean distance to compute the distance between different clusters of independent variables, merging similar clusters iteratively until only a single cluster remains. Figure 7A illustrates the same analysis process but with independent variables organized based on variable categories.

We found that certain features have the most significant negative impact on rehabilitation outcomes, such as prehospital living in intermediate care (PreHospitalLivingSetting 4) or rehab center (PreHospitalLivingSetting 9), injury at cervical level (C1-C7), diagnosis of late effects of intracranial abscess or pyogenic infection, AdmitFIMWalk-Wheelchair Measure B (both walking and using wheelchair at admission), and AdmitFIMWalk-Wheelchair Measure C (wheelchair dependent at admission). Conversely, features like lumbar SCI and intervertebral disc disorders, along with AdmitFIMWheelchair Measure W (capable of walking at admission), have the most positive correlation with rehabilitation outcomes.

Figures 6B, 7B illustrate the frequencies of inclusion of variables in the GLM models. Frequencies are determined by how often a particular feature is included (i.e., has a non-zero coefficient) across 90 prediction iterations. Yellow indicates a frequency of 1 (included in all 90 predictions), while dark green indicates a frequency of 0 (never included). The figure shows that Lasso and Elastic Net tend to select similar variables, while Ridge regression does not show variable selection in the same manner. This difference is due to the intrinsic properties of the Lasso (L1) and Ridge (L2) loss functions. The Lasso penalty forces some coefficients to shrink to zero, removing insignificant features and resulting in a sparser model. In contrast, Ridge regression compresses coefficients without forcing them to zero, leading to higher inclusion frequencies for a larger number of features. The results from Lasso and Elastic Net highlight the variables predictive of rehabilitation outcomes, such as Age, LOS, AdmitFIMBathing, AdmitFIMDressingLower, Prehospital Living Setting 4 (Intermediate Care), AdmitFIMModBowelFreqAccidents, AdmitFIMMemory, and AdmitFIMWalkWheelchair Measure C (wheelchair-dependent at admission).

**Figure 8:**
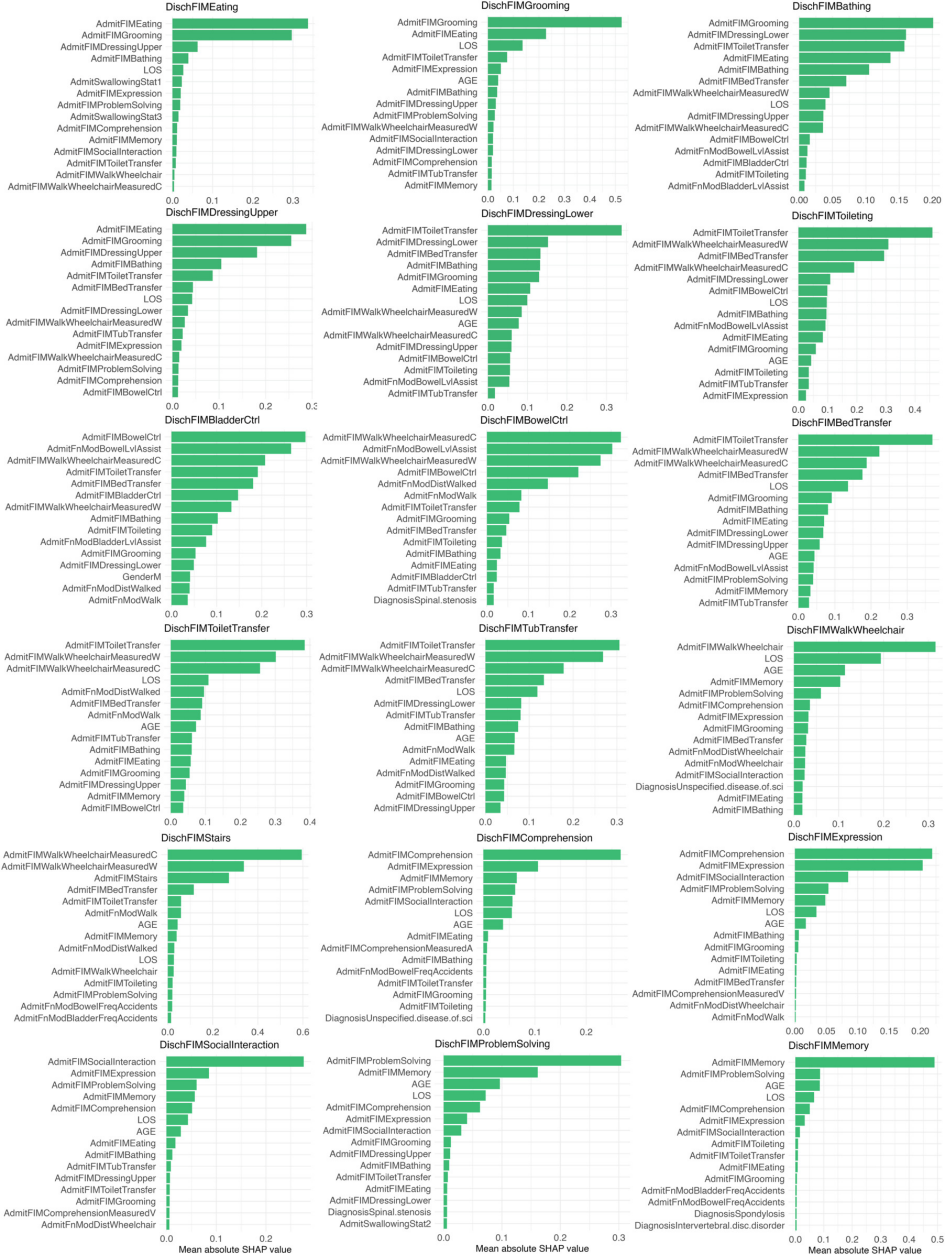
SHAP summary plots for the random forest model across 16 of the 18 FIM prediction domains. Each plot shows the features ranked by their mean absolute SHAP value, indicating their overall importance for the model's predictions for that specific outcome. The features listed at the top of each plot are the most impactful for that prediction.

To enhance transparency and provide a balanced interpretation of our models, we extended our feature importance analysis to our best-performing tree-based model. While GLMs offer direct interpretability through their coefficients, they are limited to linear relationships. To understand the drivers of our most accurate non-linear model, the Random Forest, we conducted a feature importance analysis using SHAP (SHapley Additive exPlanations) values (Figure 8). SHAP values explain the output of any machine learning model by quantifying the contribution of each feature to an individual prediction (30, 31). The analysis consistently revealed that the single most important predictor for a specific discharge FIM domain was the patient's admission score in that same domain (e.g., AdmitFIMBathing for predicting DischFIMBathing). Other features that were consistently ranked as highly important across multiple predictive domains included the length of stay (LOS) and the patient's age. This analysis complements the GLM findings and confirms the critical role of baseline functional status in predicting rehabilitation outcomes.

## Discussion

The results of this study highlight the relationships between baseline patient characteristics and long-term outcomes in SCI after rehab. By examining tree-based models and GLM models through a comprehensive grid search and subsequent evaluation, we have identified initial functional status, level of SCI, and prehospital living settings as significant predictors for rehabilitation outcomes. The consistent trends observed across both training and test datasets underscore the robustness of our findings.

The baseline model, which simply used admission scores as estimates for discharge scores, demonstrated the poorest fit, highlighting the inadequacy of simplistic predictive approaches for complex rehabilitation outcomes. Tree-based models, including Random Forest, XGBoost, and CatBoost, consistently outperformed GLMs and ordinal regression models, suggesting that these approaches are better suited to capture the complex, nonlinear interactions inherent in patients with a problem as heterogenous as SCI. In particular, Random Forest emerged as the top performing model, exhibiting the highest R-squared values, both in training and validation phases. This superior performance can likely be attributed to the model's ability to effectively partition the data and reduce variance through ensemble averaging. This underscores the necessity of employing more sophisticated models that can account for the complex nature of SCI. The performance of GLMs, while generally lower than that of tree-based models, still provided valuable insights, particularly when regularization techniques were applied. Among this class of models, Elastic Net often emerged as the most balanced approach due to its combined L1 and L2 penalties. This hybrid regularization helped in maintaining model interpretability while preventing overfitting, thereby ensuring better generalization to unseen data.

A key aspect of our analysis involved the evaluation of feature importance, particularly through the lens of GLM coefficients and their frequencies of inclusion. The hierarchical clustering of independent variables in GLM models revealed distinct patterns of influence. For instance, prehospital living in intermediate care or Rehab Center settings was found to negatively correlate with rehabilitation outcomes. This may reflect the greater baseline impairment levels, presence of comorbidities, or more complex medical needs of these patients, therefore necessitating more intensive rehabilitation efforts. It may also reflect an impact of socioeconomic status on SCI outcomes. In contrast, positive predictors of rehabilitation outcomes included lower level of injury, consistent with the current literature (32, 33). These findings suggest that current approaches to rehabilitation may be more effective for thoracic or lumbar SCI and support the idea of tailoring rehab based on specific patient characteristics in SCI. Additionally, functional measures at admission, such as the ability to walk, were strong positive predictors, a finding that is supported by prior studies (34–36). This emphasizes the critical role of initial functional status in determining recovery trajectories.

The heatmap analysis of feature coefficients in GLM models provided further granularity to our understanding. Features with high absolute values of coefficients, such as AdmitFIMWalkWheelchair and AdmitFIMBathing, consistently emerged as significant predictors. These features not only had strong individual impacts but also demonstrated high frequencies of inclusion across multiple model iterations, indicating their robustness as predictors. Tree-based models, while less interpretable in terms of individual feature importance, demonstrated superior predictive power. The ability of these models to handle interactions and nonlinear relationships without extensive preprocessing made them particularly effective in this context. The complexity and heterogeneity of SCI likely explain why such a model is necessary for good prediction of outcomes. For instance, CatBoost, with its advanced handling of categorical variables and gradient boosting approach, showed minimal overfitting and high predictive accuracy, making it an excellent choice for modeling rehabilitation outcomes.

The differences in model performance across various FIM scores also provided valuable insights into the specific areas of rehabilitation that are more predictable. Models consistently performed better in predicting outcomes related to physical transfers, such as bed and toilet transfers, compared to more complex functional areas like walking/wheelchair status and social interaction. This may be due to the more straightforward nature of physical transfers, which can be more directly influenced by rehabilitation interventions such as practicing those tasks compared to the complicated and context-dependent nature of social interactions and mobility in diverse environments.

Our analysis underscores the advantages of sophisticated modeling techniques in predicting rehabilitation outcomes. Tree-based models, with their ability to handle interactions and nonlinearities, provided the most accurate predictions, while GLMs offered valuable insights into feature importance and the relationships between predictors and outcomes. The combination of these approaches enables a comprehensive understanding of the factors driving rehabilitation success and emphasizes the value of a multi-layered approach to model selection and feature evaluation. The strong predictive power of initial functional measures suggests that timely and individualized rehabilitation plans can significantly enhance recovery trajectories. On an individual level, one of the most common questions clinicians encounter following SCI is how much function the patient will recover, if any. Our model takes steps towards answering this question and can hopefully be a part of clinical practice for counseling patients and families. More broadly, the data here suggest that patients with higher functional status after the acute phase of care from their injury tend to have the best outcomes after rehab, as would be expected.

Extending beyond individual patient care, the identification of robust predictors and high-performing models also provides guidance for resource allocation and advocates for targeted interventions that are tailored to the specific needs of patients. For example, by identifying patients who may require more intensive or specialized care, such as those living at intermediate care facilities before injury, with lower FIM scores at admission, or injured at higher spinal level, healthcare providers can allocate resources accordingly to improve the efficiency and efficacy of rehabilitation programs. By leveraging models like RF and CatBoost, clinicians can more accurately predict patient trajectories and adjust rehabilitation plans accordingly, ensuring that resources are directed towards interventions with the highest potential for impact. By integrating these findings into clinical practice, we can enhance the precision and efficacy of rehabilitation efforts, ultimately leading to better patient outcomes and more efficient use of healthcare resources.

While our machine learning models demonstrated strong predictive performance and highlighted key factors that influenced rehabilitation outcomes in our SCI patients, several limitations should be noted. First, the data were collected from a single acute rehabilitation center, which may limit the generalizability of our findings to other institutions with different patient demographics, injury characteristics, and rehabilitation protocols. Nonetheless, the large sample size and consistent data collection standard ensured strong internal validity of our results and provided the framework for future multiple-center studies for external validation. Future studies would also benefit from a prospective design to allow more precise control of potential confounding factors such as pre-injury comorbidities, injury severity, and rehabilitation intensity.

Another potential limitation is that in 2019, there was a transition from the FIM scoring system to a newer system, Section GG codes, as required by the Centers for Medicare and Medicaid Services to enhance data standardization across post-acute care settings (37). Our data collection period (2010–2015) was prior to the transition, and FIM was used to evaluate patients' functional status due to its high internal consistency and wide implementation as an SCI outcome measure in rehabilitation settings (5, 6, 38–41). Despite of the scoring system difference, the Section GG scale shares similar items with FIM, covering categories such as self-care, mobility, and cognitive function. Studies comparing FIM and Section GG have found strong positive correlations and consistency between the two scoring systems at both admission and discharge evaluations and across varying degrees of impairment (42, 43). For instance, Li et al. identified seven self-care items and six mobility/transfer items that are conceptually equivalent between FIM and Section GG (42). They compared clinician-observed scores using both systems on the same patient dataset and found that the total scores in FIM were strongly correlated with the Section GG. They also showed similar score change patterns from admission to discharge score between the two systems. Therefore, the predictive insights derived from FIM-based models are likely to remain relevant under the newer Section GG system. Moreover, our study aims to contribute to a broader understanding of how machine learning models can be implemented to predict functional outcome in SCI. The analytical framework we developed can be readily adapted to other datasets, and future work may involve retraining these models with Section GG-based data. In this way, the clinical implications of our findings extend beyond the use of FIM and remain applicable to current and evolving rehabilitation practice.

In conclusion, our study leveraged advanced machine learning techniques to predict rehabilitation outcomes for patients with spinal cord injuries and identified initial functional status, level of SCI, and prehospital living settings as significant outcome predictors. The integration of sophisticated machine learning models into both the acute setting and rehabilitation settings in SCI can facilitate more accurate predictions of patient outcomes, guiding clinical decision-making and resource allocation. This approach not only enhances patient care but also optimizes the use of healthcare resources, ensuring that interventions are directed where they are most needed. Future research should focus on refining these models and exploring additional features that may further enhance predictive accuracy. Additionally, prospective validation of the model is needed before its introduction into clinical practice. The incorporation of novel algorithms and more granular data could provide deeper insights into the rehabilitation process, paving the way for even more precise and personalized treatment plans. By continuously advancing the application of machine learning in rehabilitation, we can improve the quality of care for SCI patients and support their journey toward recovery.

## Data Availability

The raw data supporting the conclusions of this article will be made available by the authors, without undue reservation.
